# Taxonomic and Bioactivity Characterizations of *Mameliella alba* Strain LZ-28 Isolated from Highly Toxic Marine Dinoflagellate *Alexandrium catenella* LZT09

**DOI:** 10.3390/md20050321

**Published:** 2022-05-12

**Authors:** Cheng-Zhe Ren, Hui-Min Gao, Jun Dai, Wen-Zhuo Zhu, Fei-Fei Xu, Yun Ye, Xiao-Ling Zhang, Qiao Yang

**Affiliations:** 1 Department of Marine Chemistry, College of Marine Science and Technology, Zhejiang Ocean University, Zhoushan 316022, China; renchengzhe@zjou.edu.cn (C.-Z.R.); gaohuimin@zjou.edu.cn (H.-M.G.); zhuwenzhuo@zjou.edu.cn (W.-Z.Z.); 2 Natural “111” Center for Cellular Regulation and Molecular Pharmaceutics, Key Laboratory of Fermentation Engineering, Ministry of Education, College of Bioengineering, Hubei University of Technology, Wuhan 430068, China; jundai@hbut.edu.cn; 3 Zhejiang Yimeiyuan Testing Technology Co., Ltd., Zhoushan 316022, China; smexu713@163.com; 4 Zhoushan Natural Resources Surveying and Mapping Design Centre, Zhoushan 316021, China; smeye918@gmail.com; 5 ABI Group, Zhejiang Ocean University, Zhoushan 316022, China

**Keywords:** bacterial exopolysaccharides, bioflocculanting activity, microalgae growth-promoting bacterium, harmful algal bloom-forming dinoflagellate, *Alexandrium catenella*, *Mameliella alba*

## Abstract

Microalgae host varied microbial consortium harboring cross-kingdom interactions with fundamental ecological significance in aquatic ecosystems. Revealing the complex biofunctions of the cultivable bacteria of phycosphere microbiota is one vital basis for deeply understanding the mechanisms governing these dynamic associations. In this study, a new light-yellow pigmented bacterial strain LZ-28 was isolated from the highly-toxic and harmful algal bloom-forming dinoflagellate *Alexandrium catenella* LZT09. Collective phenotypic and genotypic profiles were obtained to confidently identify this strain as a new *Mameliella* *alba* member. Comparative genomic analysis showed that strain LZ-28 shared highly similar functional features with other four marine algae-derived *M. alba* strains in spite of their distinctive isolation sources. Based on the bioactivity assaying, the mutual growth-promoting effects between bacterial strain LZ-28 and algal strain LZT09 were observed. After the culture conditions were optimized, strain LZ-28 demonstrated an extraordinary production ability for its bioflocculanting exopolysaccharides (EPS). Moreover, the portions of two monosaccharides glucose and fucose of the EPS were found to positively contribute to the bioflocculanting capacity. Therefore, the present study sheds light on the similar genomic features among the selected *M. alba* strains, and it also reveals the potential pharmaceutical, environmental and biotechnological implications of active EPS produced by this new *Mameliella alba* strain LZ-28 recovered from toxic bloom-forming marine dinoflagellate.

## 1. Introduction

The term “phycosphere” was initially coined in 1972 to describe the region immediately surrounding an individual algal cell that is enriched in organic matters, which are exuded by the cell into its surroundings [1]. It is known as the aquatic analogue of the rhizosphere in the soil ecosystem [2]. The aquatic phycosphere niche is regarded as the boundary of phytoplankton holobionts and the ecological interface for algae–bacteria interactions (ABI) [3]. The relationships that usually span mutualism, commensalism, antagonism, parasitism, and competition, usually involve cross-kingdom exchanges of nutrients, secondary metabolites, infochemicals, and gene-transfer agents (GTA) [4]. It has subsequently become apparent that these microscale interactions are far more sophisticated than previously thought. It usually requires the close spatial proximity of two sides involved in the interactions, which are usually facilitated by bacterial colonization in the phycosphere niches [5]. Within this microscopic interface, the exopolysaccharides or extracellular polysaccharides (EPS) contributed by both partners are the main components of the matrix, which embeds the proliferating cells and promotes colony formation [5]. It is also the essential chemical intermedia connecting the microscale interactions [1,2,6]. Presently, it is becoming increasingly clear that these complex inter-species or intra-species associations usually exert further ecosystem-scale influences on fundamental processes including primary production, phycotoxin biosynthesis, biogeochemical cycles and the microbial loop in the oceans [7]. 

Currently, the rapidly increasing observations show that phytoplankton-associated bacterial communities are often limited to a small handful of taxa groups, including specific members of the *Roseobacter* group (*Rhodobacteraceae*), *Flavobacteraceae*, and *Alteromonadaceae* [8,9,10]. These observations also provide emerging evidence for the species-specific associations between the phytoplanktons and their closely-associated bacterial consortium [2,3,4,5,6]. *Rhodobacteraceae* usually accounts for an overwhelming proportion of the bacterioplankton communities in marine environments [8,9]. Within this family, the genus *Mameliella* has become one key and representative member in the *Roseobacter* group, although it was initially established one decade ago with *Mameliella alba* JLT354-W^T^ as the type species [11]. Moreover, *Mameliella* members widely contributed to the marine environments in aromatic compound degradation, biogeochemical cycles of carbon and sulfur, dimethylsulfoniopropionate demethylation, and the production of diverse secondary metabolites [12]. In recent decades, four type species, *Mameliella phaeodactyli*, *Mameliella atlantica*, *Ponticoccus lacteus*, and *Alkalimicrobium pacificum*, were reclassified later as heterotypic synonyms of *M. alba* based on their phylogenomic characterizations [13]. Nowadays, the genus *Mameliella* contains only two type species with correctly published names, including *M. alba* and *M. sediminis* (https://lpsn.dsmz.de/genus/mameliella, accessed on 10 May 2022). Currently, over 1500 species of free-living dinoflagellates have been described, and the harmful algal blooms (HABs)-causing group occupies over 300 species [14]. Among them, over one quarter are toxic species that produce diverse types of phycotoxins, such as paralytic shellfish-poisoning (PSP) toxins [15,16]. Within the toxic dinoflagellate species, the globally distributed genus *Alexandrium*, in which the members were widely found in sub-polar, temperate and tropical coastal water environments, is a particularly well-known bloom-forming group due to its large and widespread threat to seafood production and human health [17,18]. 

The importance of the phycosphere has been postulated for four decades, yet only recent new technological progress in high-throughput pyrosequencing made it possible to start teasing apart the complex nature of the microbial composition within this unique microbial habitat [3,4,5,6,19,20,21,22,23,24]. The culture-independent isolation of the cultivable strains is still the first critical step for exploring the dynamics of algae–bacteria interactions [3,4,5,6]. Previously, we conducted the Phycosphere Microbiome Project (PMP) to convey the compositions of varied microbial consortiums that were closely-associated with diverse HAB-forming dinoflagellates [25,26,27,28,29,30]. During our investigation, the genus *Mameliella* was found to be one predominant group and was widely distributed in the bacterial communities associated with various marine dinoflagellates [10,19,20,21,22,23]. Consequently, a new red-pigmented bacterial strain, LZ-28, was isolated from the highly toxic *Alexandrium catenella* LZT09. Strain LZ-28 produced active bioflocculanting exopolysaccharides, which were also discovered in other cultivable bacterial strains isolated from marine dinoflagellates [27,28,29,30,31]. Additionally, it demonstrated the obvious growth-promoting ability of the algal strain LZT09, as previously verified by other marine bacteria strains derived from the same algal host [21,30]. Thus, the present work aims to characterize the taxonomic status of this new bacterial strain using combined taxonomic and phylogenomic approaches. Moreover, comparative genomic analysis is then emphatically performed for the five marine algae-derived strains among the ten selected *M. alba* strains to reveal their shared functional nature. Based on bioactivity assaying after culture condition optimization, the contribution of the individual monosaccharide portion of the EPS produced by strain LZ-28 to the bioflocculanting bioactivity is also characterized. 

## 2. Results and Discussion

### 2.1. Characterization of the Composition of the Bacterial Community of Algal Strain LZT09 

**Figure 1:**
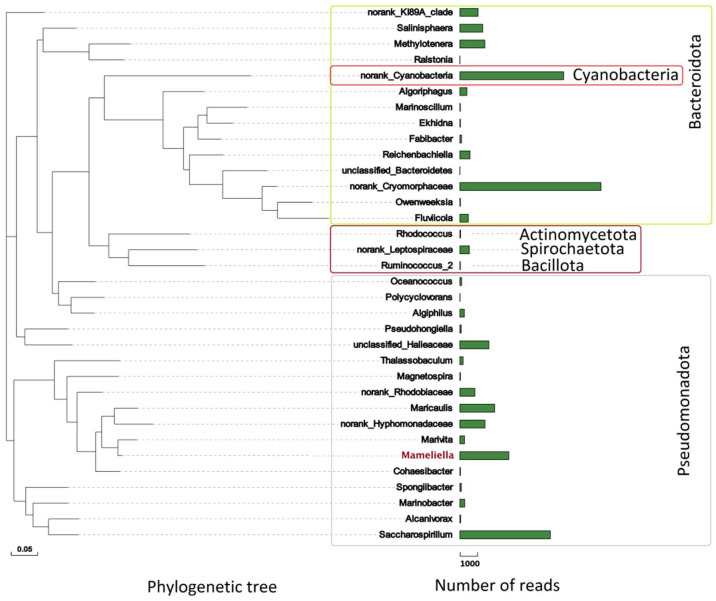
Phylogenetic tree and the number of reads of bacterial abundance at the genus level of the bacterial community associated with algal strain LZT09 by high-throughput sequencing of the V3-V4 variable region of bacterial 16S rRNA gene. Bar: 0.05 substitutions per nucleotide position in the phylogenetic tree.

To reveal the microbial composition of the bacterial community associated with LZT09, the high-throughput sequencing of the V3–V4 variable region of bacterial 16S rRNA was performed. Based on the obtained result, the phylum Pseudomonadota represented the overwhelming dominant bacterial fraction (53.9%) of the total bacterial community of LZT09, followed by Bacteroidota (26.7%) and Cyanobacteria (15.2%). Other lineages, including Actinomycetota, Bacillota, and Spirochaetota, were also present, but each constituted less than 1.0% of the total (Figure 1). Similarly, previous studies showed that two phyla, Pseudomonadota and Bacteroidota, were the most major bacterial groups affiliated with the investigated bacterial community associated with marine dinoflagellates [20,21]. Moreover, members of *Rhodobacteraceae* were reported to be frequently present during various stages of HABs [32,33], and also regarded as a major taxa associated with some diatom cultures [34,35]. 

In this study, *Rhodobacteraceae* was found to occupy only 8.7% of the total bacterial community of LZT09, which held the fifth dominant group following the family *Cryomorphaceae* (22.9%), one unidentified Cyanobacteria (16.8%), *Saccharospirillaceae* (14.7%), and the *Hyphomonadaceae* (9.7%). *Cryomorphaceae* is a member of the order Flavobacteriales within the phylum Bacteroidota, and has been found to distribute in a wide range of marine and terrestrial systems from tropical to polar regions [36]. Moreover, molecular phylogenetic studies show that the phylotypes related to the family *Cryomorphaceae* belong to the abundant coastal phytoplankton bloom-responding flavobacterial group [37]. In addition, at the genus level, five dominant bacterial groups, including one unidentified *Cryomorphaceae* (22.9%), one unidentified Cyanobacteria (16.8%), *Saccharospirillum* (14.6%), *Maricaulis* (5.6%), and *Mameliella* (5.1%), were identified, and totally accounted for 65.0% of the total bacterial community of LZT09 (Figure 1). It is worth noting that only one genus, *Microcystis*, was found in the Cyanobacteria group, which indicated that some cryptic cyanobacterial lineages were potentially hidden by the dinoflagellate host [37].

### 2.2. Phenotypic and Biochemical Characteristics of Bacterial Strain LZ-28

**Figure 2:**
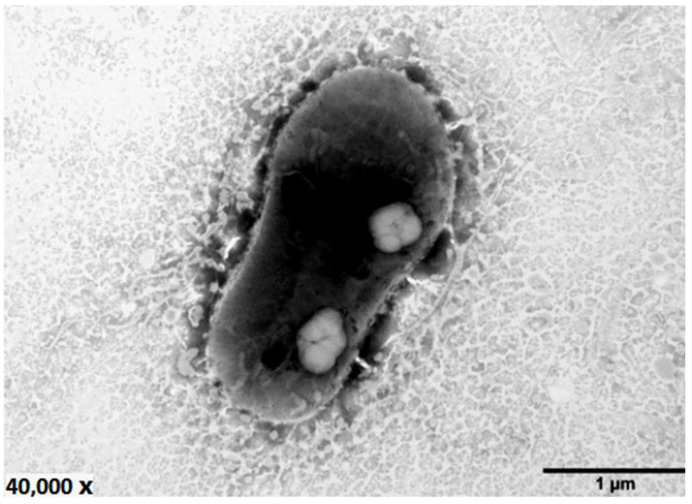
Transmission electron micrograph of the cell of the bacterial strain LZ-28 after being grown on MA at 28 °C for 2 days. Bar: 1 μm.

Previously, nine cultivable bacterial strains, including strain LZ-28, were isolated from the cultivable PM of LZT09 [10]. However, no strain was found to belong to the dominant group *Cryomorphaceae.* Previous studies have shown that the most cultured *Cryomorphaceae* species were mainly isolated from the low-temperature ecosystems, although the family *Cryomorphaceae* was widespread in a wide range of non-extreme ecosystems. These findings indicate the resistance of the *Cryomorphaceae* members towards be cultivation [36]. Thus, the constant optimization of the isolation procedure for the cultivable bacteria strains isolated from marine dinoflagellates still needs to be performed, especially by means of the emergence and prevalence of modern multi-omics data [38,39]. Based on the phenotypic characterization, the cellular colonies of strain LZ-28 grown on marine agar (MA) were circular, smooth, and convex with light-yellow colors. Cells of strain LZ-28 were Gram-stain-negative, rod shaped, and non-motile with the sizes of 0.7–1.0 μm for the width and 2.0–2.9 μm for the length (Figure 2). 

The physiological characterization revealed that strain LZ-28 grew at the pH ranging from 5.0 to 10.0 with the optimum growth at pH 7.0. The growth temperature ranged from 15 °C to 40 °C with the optimum value of 25–28 °C, at the presence of 1–10% (*w/v*) NaCl with the optimum value of 2.5%. No bacterial growth was observed under anaerobic conditions when grown on MA, even after three week-long incubations. The hydrolysis of starch, L-tyrosine, and gelatin were observed. Nitrate was reduced to nitrite, but the reduction of nitrite to nitrogen was not observed. Chemotaxonomic analysis showed that the major cellular fatty acids of strain LZ-28 consisted of C_18:0_, C_18:1_ω7c 11-methyl, and C_19:0_ cyclo. Detailed comparisons of the morphological, biochemical, and physiological characteristics of strain LZ-28 and the other five *M. alba* strains are summarized in Table 1. The differential characteristics between strain LZ-28 and other *M. alba* members can be easily found, even though they share very high 16S rRNA gene-sequence similarities, for example, the distinguished colony color, the ability for citrate utilization, the presence/absence of a minor fatty acid of C_17:1_ω8c, as well as the apparently varied polar lipid profiles among the six compared *M. alba* strains.

### 2.3. Phylogenetic Analysis Based on 16S rRNA Gene Sequences

**Figure 3:**
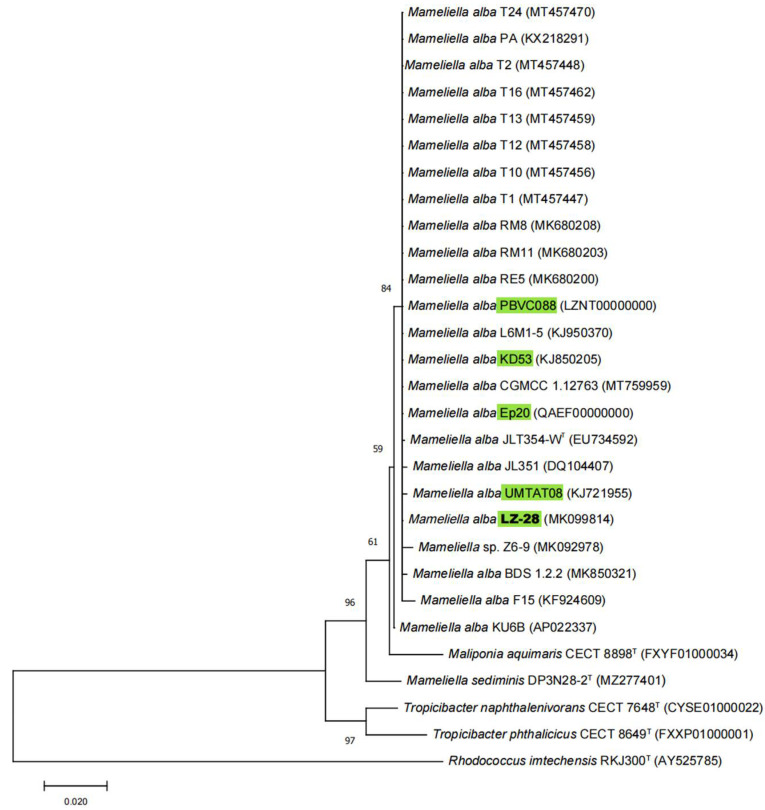
Phylogenetic tree using the maximum-likelihood (ML) algorithm constructed based on 16S rRNA gene sequences showing the relationship between *Mameliella* strains and other representative type species in the family *Rhodobacteraceae*. Bootstrap values (≥50%) based on 1000 replications are shown at the branch points. The five selected *M. alba* strains isolated from marine algae are marked in green. *Rhodococcus imtechensis* RKJ300^T^ (AY525785) was used as an outgroup. *Bar*, 0.02 substitutions per site.

In the phylogenetic tree constructed by the maximum-likelihood (ML) algorithm using the 16S rRNA gene sequences, strain LZ-28 formed a monophyletic branch together with the type and other non-type strains of *M. alba*, as well as the selected representative *Rhodobacteraceae* members (Figure 3). Previously, *M. phaeodactyli* (type-strain KD53) [12], *M. atlantica* (type-strain L6M1-5) [40], *Ponticoccus lacteus* (type-strain JL351) [41], and *Alkalimicrobium pacificum* (type-strain F15) [42] were later reclassified as heterotypic synonyms for *M. alba* based on the phylogenomic characterizations [13]. In this study, the phylogenetic analysis showed that strain LZ-28 shared high 16S rRNA gene similarity values of 99.70, 99.77, 99.92, 99.60, and 99.40% with five other *M. alba* strains, JLT354-W^T^; KD53; L6M1-5; JL351; and F15, respectively, although they were obviously isolated from different sources (Table 1). These values were all exceeding the threshold values (98.65%) generally accepted for species delineation [43]. Thus, it collectively indicated that strain LZ-28 was affiliated with the genus *Mameliella*, and was probably a new member of *M. alba*.

### 2.4. Genomic Features of the Selected M. alba Strains

Due to the high-similarity values of the 16S rRNA gene sequence between strain LZ-28 and its phylogenetic neighbors in the family *Rhodobacteraceae*, the extra phylogenomic characterization was performed to ensure its taxonomic status. The genome sequence of strain LZ-28 was obtained by our previous study [10], and the other nine *M. alba* strains with available genomes were obtained from the NCBI database (https://www.ncbi.nlm.nih.gov, accessed on 2 April 2022). The isolation sources and the general genomic characteristics of the ten selected *M. alba* strains are summarized in Table 2. Among the ten selected *M. alba* strains, only strain KU6B has a complete genome containing a 5.386 Mbp circular chromosome and three circular plasmids of 256, 112, and 76 Kbp, respectively [44]. The genomic size of strain LZ-28 was 5.66 Mb with a G+C content of 64.94%. It contained 5497 protein-coding DNA sequences (CDSs) and 61 RNA genes, including 8 rRNA, 50 tRNA, and 3 other RNA genes, respectively, as well as 86 pseudogenes. The genome of strain LZ-28 was the fifth largest, with a genome range of 5.26–5.90 Mb. However, the genomic DNA G+C content of strain LZ-28 was the lowest (64.94 mol%) among the ten selected *M. alba* strains. 

### 2.5. Phylogenomic Characterization of Bacterial Strain LZ-28

**Figure 4:**
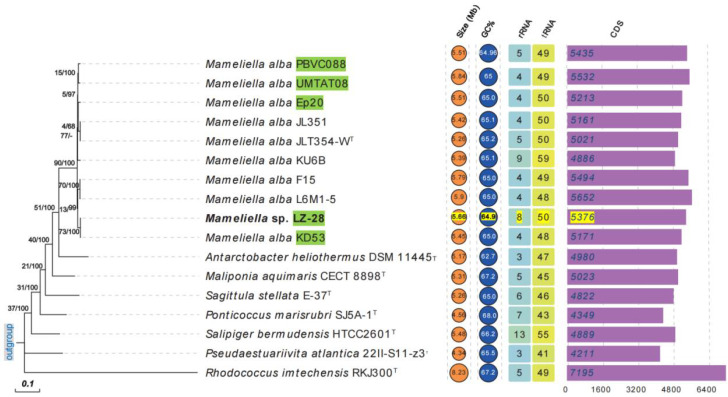
Phylogenomic tree constructed by up-to-date bacterial core gene (UBCG) set among the ten selected *M. alba* strains, as well as representative type species in the family *Rhodobacteraceae*, and a comparison of the five general genomic characteristics, including the genomic size, DNA G+C content (%), and numbers of predicted protein-coding genes (CDS, rRNA, and tRNA). The gene-support index (the number of individual gene trees presenting the same node for the total genes used) (**left**) and bootstrap values (**right**) were presented at the nodes of the phylogenomic tree. The five selected *M. alba* strains isolated from marine algae are marked with green, and the genome parameters for strain LZ-28 were marked with yellow. *Bar*: 0.1 nt substitutions per site.

To further infer the phylogenetic relationship among the ten selected *M. alba* strains with available genome sequences, the phylogenomic tree using an up-to-date bacterial core gene set (UBCG) consisting of 92 bacterial core genes was also performed. The constructed phylogenetic tree is shown in Figure 4. It clearly shows that, among the five marine algae-derived *M. alba* strains, strain LZ-28 clusters together with strain KD53, which is isolated from marine diatom *Phaeodactylum tricornutum* [12]. The other three strains, PBVC088, UMTAT08, and Ep20, were also clustered together in the UBCG tree. Two clusters were also observed in the phylogenomic tree. One cluster was composed of strains JLT354-W^T^, JL-351, and KU6B, which were all isolated from the surface seawater, and the second cluster included two strains, L6M1-5 and F15, which were both isolated from the deep-sea sediment. It indicated that the genome-based phylogeny plays a more definitive role in the construction of a natural and objective taxonomy [43]. Additionally, the comparison of the three key phylogenomic parameters, including average nucleotide identity (ANI, Figure S1A), average amino acid identity (AAI, Figure S1B), and digital DNA–DNA hybridization (dDDH, Figure S1C) values of strain LZ-28 and the type strain of *M. alba* JLT354-W^T^ were 98.0%, 98.4%, and 84.3%, respectively. All the values were higher than the thresholds (95–96% for ANI, 97% for AAI, and 70% for dDDH) generally accepted for new species delineations [43]. Accordingly, it clearly confirmed that strain LZ-28 was a new member of *M. alba*, based on the obtained taxogenomic evidences.

### 2.6. Comparison of the Core- and Pan-Genomic Profiles among the Selected M. alba Strains

**Figure 5:**
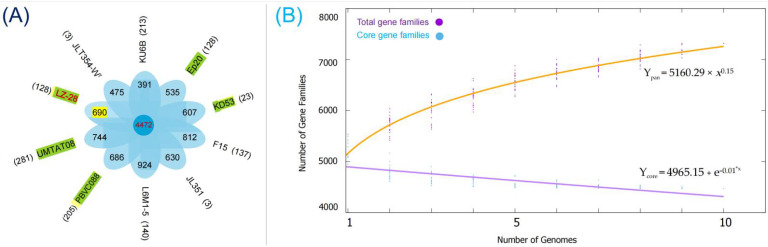
Core–pan-genome comparison of the ten selected *M. alba* strains based on the bacterial pan-genome analysis (BPGA) pipeline. (**A**) Petal diagram of the core, accessory, and unique genes of the pan genome. The number of core genes is displayed in the central circle. Overlapping regions represent the number of accessory genes. Strain names are marked outside each petal, along with the strain-specific gene number shown in the brackets. The five selected *M. alba* strains isolated from marine algae are marked with green. (**B**) Accumulation curves for the pan and core genomes of the ten *M. alba* strains. The purple and blue dots represent the number of pan- and core genes, respectively. The equations are illustrated in each curve.

The genome sequences of the other nine selected *M. alba* strains were retrieved from the NCBI database in addition to strain LZ-28. The numbers of accessory and unique genes of the ten *M. alba* strains were created and shown in Figure 5A. It shows that 4472 core genes are shared by the ten selected *M. alba* strains, which account for 80.8% to 90.3% of the genome repertoire of each strain. Additionally, only 0.1% to 5.1% of the unique genes were distributed in individual strains, depending on the varied genomic sizes. Two functional accumulation curves were constructed according to the core- and pan-genome analyses, respectively. The pan genome of the ten *M. alba* strains was fitted into a power law function with an exponent γ = 0.15 [Fp(*x*) = 5160.29 × *x*^0.15^], and did not appear to reach saturation even with the increasing genome number. Thus, it indicated that the pan genome was in an open state. This kind of open pan genome often exists in bacterial species residing in multiple ecological environments, and has multiple ways of exchanging genetic material through horizontal gene transfer (HGT) [45]. The core genome was fitted into an exponential regression [Fc(*x*) = 4965.15 + e^−0.01*x*^]. Based on the two constructed functional curves, the gene number of the pan genome increased when the species number of the *M. alba* strain accumulated. Meanwhile, the tendency of the core genome, on the contrary, implied that more strains tended to result in additional numbers of unique genes.

**Figure 6:**
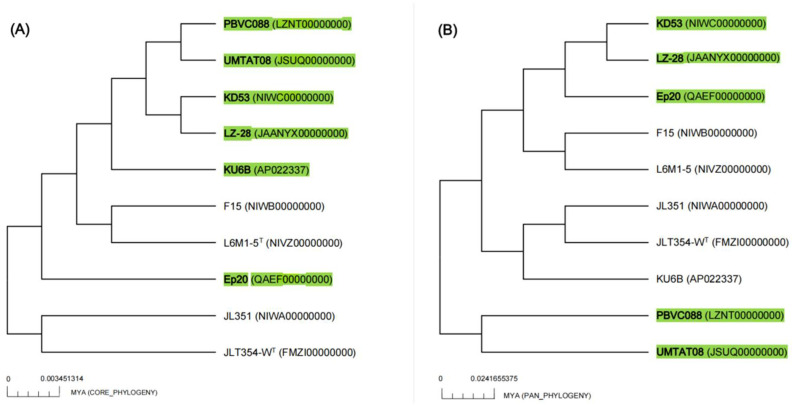
Phylogenetic trees constructed by core (**A**) and pan genomes (**B**) of the ten selected *M. alba* strains inferred from maximum likelihood (ML) analysis using concatenated alignment of 303 pan-orthologous genes. The estimated divergence time from present (million years ago; MYA) is given as the bar for both trees. The five bacterial strains isolated from marine algae are marked with green.

The phylogenetic trees of the core and pan genomes of the ten selected *M. alba* were constructed and shown in Figure 6. It indicates the highly conservative evolution among the *M. alba* members investigated in this study. The obvious difference of the number of unique genes among the ten selected *M. alba* strains may be explained by their adaptions to the growth conditions and possible horizontal gene-transfer events [45]. Moreover, strain LZ-28 showed a close phylogenetic relationship with the marine algae-derived strains, either with strains KD53 and UMTAT08 in the core-genome phylogenetic tree (Figure 6A), or with strains KD53 and Ep20 in the pan-genome phylogenetic tree (Figure 6B). Thus, it indicates the genome-based phylogenetic analysis provides robust evidence for the evolutionary history of the individual *M. alba* strain [46].

### 2.7. Comparison of the Functional Classes of Predicted Genes among the Selected M. alba Strains

The functions of gene families in the genomes of the ten selected *M. alba* strains were evaluated by performing the COG and KEGG categories analyses. The COG distribution profile showed that most core (Figure S2A) and unique (Figure S2B) genes were both mainly related to five functional groups, including carbohydrate transport and metabolism [G], transcription [K], replication, recombination and repair [L], general function prediction only [R], and unknown function [S]. Three strains, including strain KU6B isolated from surface seawater, strain KD53 from marine diatom *Phaeodactylum tricornutum* [12], and strain UMTAT08 from marine dinoflagellate *Alexandrium tamiyavanichii* [47], harbored the largest core gene number among the ten selected *M. alba* strains during COG analysis (Figure S2A). Moreover, most unique genes were found in strains KU6B and UMTAT08 (Figure S2B). With respect to the KEGG assignment, based on the characterization of the core and unique genomes, the genes related to general function, amino acid metabolism, and carbohydrate metabolism, accounted for the major types of KEGG categories. In addition, two strains, UMTAT08 and F15, harbored the largest unique gene number among the ten selected *M. alba* strains during COG analysis (Figure S2D). Additionally, no unique gene sorted in COG and KEGG categories was observed in strain JL351 isolated from the surface seawater [41]. 

For the core-gene groups of the COG category, the five marine algae-derived *M. alba* strains demonstrated a more scattered distribution pattern (Figure S2A). However, for the unique gene groups of COG, the principal components analysis (PCA) showed two obviously separated groups among the five marine algae-derived *M. alba* strains. One group consisted of strains Ep20 and KD53, and the second group included strains LZ-28 and PBVC088. For strain UMTAT08, isolated from *A. tamiyavanichii* [47], it was far away from the two clustered groups (Figure S2B). In addition, based on the two PCA analyses of the core- (Figure S2C) and unique-KEGG (Figure S2D) profiles, it was highly noteworthy that strain LZ-28 both showed a functional relationship similar to the other three marine algae-derived *M. alba* strains, except for strain UMTAT08. Thus, it indicated that the four selected marine algae-derived *M. alba* strains shared similar functional destinations in spite of their distinctive isolation sources.

### 2.8. Growth-Promoting Effects of Bacterial Strain LZ-28 and Algal Strain LZT09

**Figure 7:**
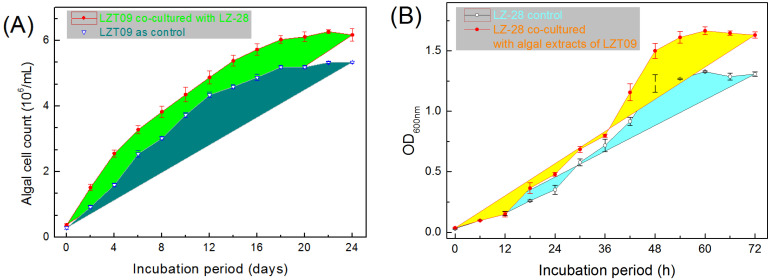
The microalgae growth-promoting potential of the bacterial strain LZ-28 on algal strain LZT09 (**A**), and the promoting effect of algal extract of LZT09 on the bacterial growth by the measurements of the optical changes recorded at OD_600 nm_ (**B**).

Based on the microalgae growth-promoting (MGP) assay [21], strain LZ-28 demonstrated obvious growth-promoting activity when co-cultured with algal host LZT09 (Figure 7A). Interestingly, the mutual promoting effect of the algal culture extract from LZT09 on the bacterial growth of strain LZ-28 was also observed (Figure 7B). It indicated that the potential association between bacterial strain LZ-28 and algal strain LZT09 in spite of the detailed nature of action mechanism was still unclear. Furthermore, deeply unearthing the multi-omics information for both sides in co-culture circumstances is believed to offer substantial insights into the detailed mechanism governing these dynamic interactions [48,49,50,51,52].

### 2.9. Optimization of Bacterial Growth and EPS Accumulation 

**Figure 8:**
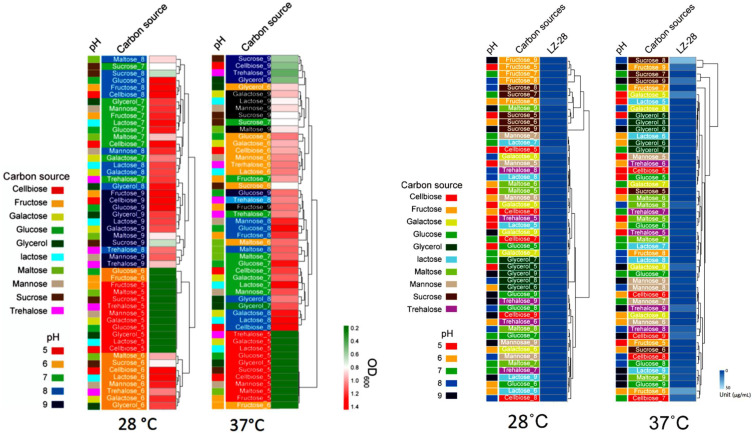
Comparison of the effects of the ten carbon sources and the pH range (5.0–9.0) of the media on bacterial growth (**left**; OD_600_) and EPS accumulation (**right**; unit, μg/mL) of strain LZ-28 cultured at 28 °C and 37 °C, respectively.

Bacterial exopolysaccharides (EPSs) were revealed to serve as one essential chemical intermedia within the microscopic phycosphere niches and mediate host–microbe interactions [2,3,4,5,53]. During our previous investigations, several novel bacterial species, which produce bioactive EPSs, were also recovered from the freshwater [26] and marine phycosphere [27,28,29,30,31], as well as the gut microbiota of Antarctic emperor penguin [48,54,55]. In this study, the preliminary experiment showed that temperature and carbon sources were the main two factors influencing the bacterial growth and EPS accumulation. Therefore, ten carbon sources, including cellbiose, fructose, galactose, glucose, glycerol, lactose, maltose, mannose, sucrose, and trehalose, and the pH range of 5.0–9.0 were further used for the optimization of the culture conditions. Based on the obtained result, bacterial incubation at 37 °C promoted bacterial growth and shortened the incubation period of strain LZ-28, although the general trends of bacterial growth that were cultured at 28 °C and 37 °C demonstrated a similar pattern. For the pH tests, strain LZ-28 grew better in the pH values ranging from 6.0 to 9.0. Additionally, strain LZ-28 was found to achieve the fastest growth rate when the cellobiose was used as a carbon source, and cultured at 28°C or at 37 °C and at pH 7.0 (Figure 8). Based on the EPS accumulation measurements, when cultured at 28 °C, the higher EPS yield of strain LZ-28 was achieved both at pH 5.0 and pH 6.0. However, when the culture temperature was changed to 37 °C, the EPS yields were observed to gradually enhance with the increasing pH values, and reached the maximum at pH 9.0. Under the optimized conditions, the highest EPS yield of 47.7 μg/mL was obtained when sucrose (10 g/L) was used as a carbon source and cultured at 37 °C and at pH 9.0 (Figure 8).

### 2.10. Bioflocculanting-Activity Evaluation and Correlation Analysis 

**Figure 9:**
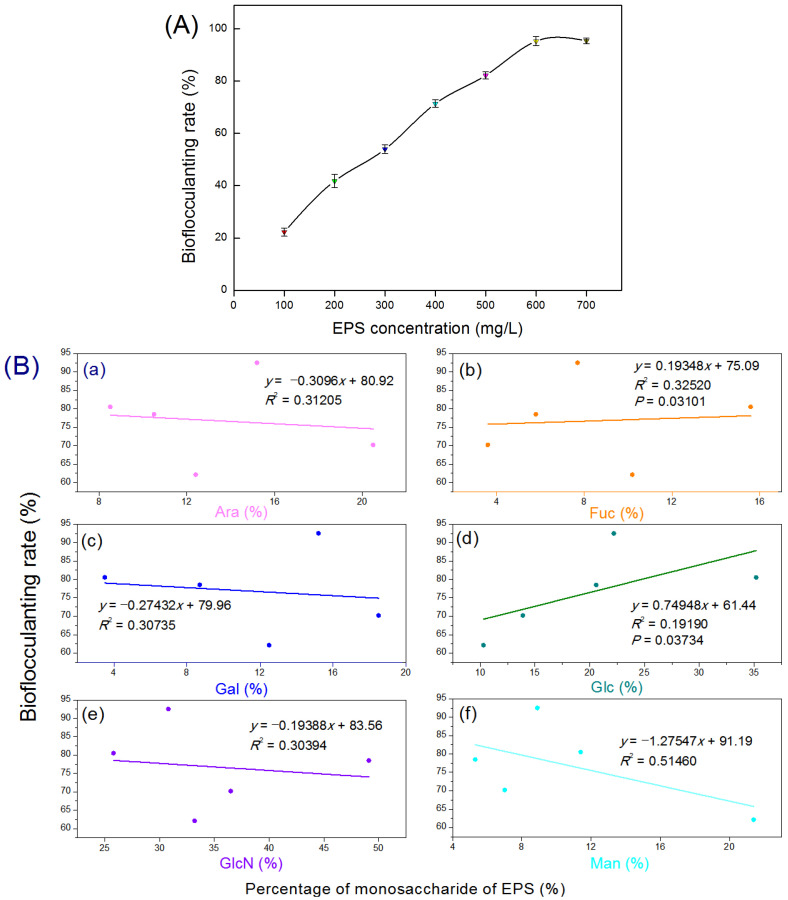
Bioflocculanting activities of the bacterial exopolysaccharides (EPSs) produced by bacterial strain LZ-28 (**A**), and correlation analysis (**B**) of the relative portions (%) of the six monosaccharides including arabinose (Ara, pane **a**), fucose (Fuc, pane **b**), galactose (Gal, pane **c**), glucose (Glc, pane **d**), amino-glucose (GlcN, pane **e**) and mannose (Man, pane **f**) of the bacterial EPS with the flocculanting efficiency expressed with the bioflocculanting rate (%).

Based on the bacterial bioflocculanting assaying, EPS extracted from strain LZ-28 demonstrated obvious bioflocculanting activity, which showed a concentration-dependent manner (Figure 9A). The highest efficiency of the bioflocculanting rate of 95.7 ± 8.5% was achieved when 0.60 g·L^−1^ of EPS was applied. It exhibited a higher bioflocculanting capacity compared to the other bacterial strains, which were also recovered from the marine dinoflagellates previously reported [27,28,29,30,56,57,58,59]. To further infer whether the type and abundance of the monosaccharides of the polymer EPSs were related to the bioflocculanting activity, the characterizations of the monosaccharide compositions of the EPSs and their correlations with the bioflocculanting activity were then performed. Base on the obtained result, the relative portions of two monosaccharides, glucose and fucose, in the crude polysaccharides demonstrated significantly positive highly strong correlations (both *p* < 0.05) with the bioflocculanting capacities with the correlation coefficients of 0.9 (*p* = 0.03734) and 0.8 (*p* = 0.03101), respectively, whereas the other four monosaccharides were found to be negatively contributed without statistical significance (Figure 9B). Despite these findings, the detailed chemical structures of the EPSs produced by strain LZ-28 remain to be further elucidated. Moreover, more experimental data are still needed to dig out reliable clues to explore the structure–activity relationship of the bacterial EPSs as promising and natural microbial bioflocculants. In addition, the genomic mining also revealed the presence of several typical biosynthesis genes (wzx, exo, and muc) for bacterial EPS biosynthesis in strain LZ-28. Thus, the present study indicated that strain LZ-28 could serve as a new, fresh bacterial candidate with natural potential for the production of versatile EPS bioflocculants derived from marine microalgae with potential pharmaceutical, environmental, and biotechnological implications [27,28,29,30,55,56,57,58,59,60,61]. 

## 3. Materials and Methods

### 3.1. Algal and Bacterial Strains and Culture 

*Alexandrium catenella* LZT09 was sampled in the Zhoushan Archipelago area in the East China Sea during an algal bloom occurrence in July 2018, and then routinely cultured in our ABI laboratory [10]. LZT09 produced high levels of paralytic shellfish-poisoning (PSP) toxins [21]. LZT09 cells were cultured for 21 days in 50 mL of F/2-enriched seawater [21], and were exposed to a 12 h light–dark cycle at 25 °C (ca. 125 μmol photons m^−2^ s^−1^). Algal cells were obtained by centrifugation (4000× *g* for 20 min) and the resultant cell pellet was stored at −20 °C until used. The axenic LZT09 was obtained by repeated washing, lysozyme/SDS and antibiotic treatment according to the procedure previously reported [62]. For comparative purposes, five strains, including *Mameliella phaeodactyli* KD53 (MCCC 1K00273), *Mameliella* *atlantica* L6M1-5 (MCCC 1A07531), and *Alkalimicrobium pacificum* F15 (MCCC 1A09948), purchased from the Marine Culture Collection of China (MCCC), and strains *Ponticoccus lacteus* JL351 (CGMCC 1.12986) and *Mameliella* *alba* JLT354-W^T^ (CGMCC 1.7290^T^) purchased from the China General Microbiological Culture Collection Center (CGMCC) were used. All the tested strains were grown in the same conditions for the experiments.

### 3.2. PCR Amplification, 16S rRNA Sequencing, and Data Analysis

Total genomic DNA (gDNA) was extracted using a Fast Bacterial Genomic DNA Extraction Kit (Sangon Biotech Co., Ltd., Shanghai, China) according to the manufacturer’s instructions. A general bacterial primer pair 338F/806R was used to amplify the V3–V4 variable region of the bacterial 16S rRNA gene [19,20]. The 16S rRNA sequencing libraries for Illumina library was constructed using the TruSeq^®^ DNA PCR-Free Sample Preparation Kit (Illumina, San Diego, CA, USA), then sequenced using a NovaSeq 6000 PE250 platform utilized at BioMajor (Shanghai, China). To analyze the microbial community using the obtained Illumina sequencing data, the script from QIIME2 (https://qiime2.org, accessed on 10 May 2022) was used [63]. The paired-end reads were joined using the multiple_join_paired_ends.py script and quality filtered by the multiple_split_libraries_fastq.py script. The obtained OTUs were clustered using UCLUST with a default threshold of 0.97 [64]. The OTU phylotypes were assigned using the closed-reference OTU picking method against the QIIME modified version of the SILVA database, v128 [65]. The parameters used in the above analysis were all default settings. 

### 3.3. Morpho-Physiological and Biochemical Characterizations

The preparation of the transmission electron microscopy (TEM) sample was performed using the negative-staining method [54,55]. Briefly, vegetative cells of strain LZ-28 were observed from a fresh, 24 h culture inoculated in MB. The cells were fixed in 2.5% glutaraldehyde in a phosphate buffer (0.1 M; pH 7.2) for 2 h at 4 °C, then washed with the same phosphate buffer post fixed with 1.0% OsO_4_. The obtained samples were dehydrated in serial 30–100% ethanol, then mounted on copper grids, and observed using a JEM-1200 TEM instrument (EOL, Tokyo, Japan). Gram-staining test was followed by the our procedures previously described [56,57,58]. The hanging-drop technique was used for observing motility during the exponential phase of the culture growth. The growth conditions of temperature were tested using MA medium at the temperature ranges of 4, 10, 15, 20, 25, 28, 30, 33, 37, 40, 45, 50, and 55 °C. The optimal pH was investigated at different pH levels ranging from 4.0 to 11.0 with 0.5 unit intervals and cultured in MB at 28 °C for 2 days. Salt tolerance was determined by growth in an MB medium containing various NaCl concentrations (0–15.0%, w/v, with interval of 0.5%) while removed the original NaCl [47,48]. Catalase and oxidase activities were detected by adding 3% (*v/v*) H_2_O_2_ and using oxidase reagent (bioMérieux, Marcy-l’Étoile, France), respectively. Anaerobic growth was assessed after 1 week of cultivation in an anaerobic chamber (Bactron EZ-2; Shellab, Cornelius, OR, USA) on MA at 28 °C. Tests of phenotypic and enzymatic characterizations were conducted using API 20NE, API 20E, and API ZYM strips (bioMérieux) according to the manufacturer’s instructions.

### 3.4. Analysis of Fatty Acid Profiles

For whole-cell fatty acid analysis, strain LZ-28 and the five reference strains were grown on MA at 28 °C for 2 days. Sufficient cells of comparable physiological ages were harvested from the third streak quadrant of the agar plates and cellular fatty acids were saponified, methylated, and extracted using the standard protocol of the Sherlock Microbial Identification (MIDI) System (Sherlock version 6.1, Newark, Delaware, USA). Fatty acid methyl esters were analyzed by an Agilent 6890N gas chromatography system (GC) and identified using the MIDI TSBA6 database [66]. 

### 3.5. Phylogenetic Analysis Based on the 16S rRNA Gene Sequences

The related 16S rRNA gene sequences of species in the genus *Mameliella* and type strains in the family *Rhodobacteraceae* were retrieved from the NCBI database, except strains PBVC088 and Ep20 [67], which were extracted from the genome sequences by PhyloSuite version 1.2.1 [68]. The identification of phylogenetic neighbors and the calculation of pairwise 16S rRNA gene sequence similarities were achieved using the EzTaxon-e server (http://eztaxon-e.ezbiocloud.net, accessed on 2 April 2022). The sequence alignments were performed with CLUSTAL_X (http://www.clustal.org, accessed on 2 April 2022) [69]. Phylogenetic trees were reconstructed with the maximum-likelihood (ML) algorithm [70] by the Tamura Nei model [71] in the software package MEGA version 7.0 (https://www.megasoftware.net, accessed on 2 April 2022) [72]. The resultant tree topologies were evaluated by bootstrap analyses [73] based on 1000 re-samplings.

### 3.6. Phylogenomic Calculations and UBCG Tree Construction

The draft genome sequence of LZ-28 was obtained by our previous investigation [10]. The reference genome sequences of the *Mameliella* *alba* strains, six *Rhodobacteraceae* strains, and one *Rhodococcus* strain were retrieved from the NCBI database. The average nucleotide identity (ANI) and the average amino acid identity (AAI) analyses were performed using the ANI/AAIMatrix genome-based distance matrix calculator (http://enve-omics.ce.gatech.edu/g-matrix, accessed on 19 March 19 2022) and the digital DNA–DNA hybridization (dDDH) analysis was conducted using the Genome-to- Genome Distance Calculator (GGDC 2.1) (http://ggdc.dsmz.de/, accessed on 19 March 2022) using Formula 2 [74]. The phylogenetic tree based on the 92 bacterial core genes was constructed by using the UBCG pipeline [75]. Further visualization and editing of the UBCG tree were accomplished by EvolView tool (https://www.evolgenius.info/evolview, accessed on 19 March 2022). Phylogenetic trees based on pan genes and 4472 bacterial core genes were conducted by the bacterial pan-genome analysis (BPGA) pipeline v.1.3 [76] using USEARCH with the default settings [77].

### 3.7. Pan-Genome Analysis

The BPGA pipeline was used to automate the complete pan-genome study and downstream analysis of the prokaryotic sequences [69]. All 11 protein sequences annotated by Prokka [78] were used for the BPGA (sequence identity ≥50%; and E value ≤1.0 × 10^−5^) tool to perform the core–pan-genome analysis. The genomic accumulation curve was generated by the BPGA’s result via gnuplot. The core, accessory, and unique genes were classified from the orthologous groups through the USEARCH clustering algorithm [77]. 

### 3.8. Comparative Analysis of Functional Genes 

The online COG and KEGG databases accessed via BPGA were used to analyze the functions of the genes. The core, accessory, and unique genes were sorted, and the major concerns related to the core and the unique genes. The gene clusters were identified by using anti-SMASH 6.0.0 [79] from the reference genome sequences of *M. alba* KD53. All 11 protein sequences produced by Prokka were used for the eggnog mapper to obtain an overview of the functional genes [80]. All results were manually checked. The principal component analysis (PCA) was performed by using CANOCO 5 (http://www.canoco5. com/index.php/resources, accessed on 19 March 2022) to compare the correlations across all samples based on the relative abundance of the function genes. The Spearman’s rank correlations were determined by using the SigmaPlot program version 11.0 (Systat Software, Inc., San Jose, CA, USA). 

### 3.9. Bacterial Growth and EPS-Accumulation Analysis

For the culture optimization experiment, 1 mL of 24 h fresh bacterial culture of strain LZ-28 was obtained and mixed with 24 mL of fresh 2216 medium, then 150 μL of the mixture was added into the wells of the 96-well microplate containing different carbon sources and cultured with shaking at 60 rpm/min for 72 h. During the culture period, the bacterial growth rate was monitored by the measurement of the optical density change recorded at OD_600nm_ every 3 hours. A total of 10 carbon sources, including cellobiose, fructose, galactose, glucose, glycerol, lactose, maltose, mannose, sucrose, and trehalose, added with the final concentration of 10 g/L, and the pH range of 5.0–9.0 with 1.0 as the interval were used for the measurements cultured either at 28 °C or 37 °C. The extraction and the quantification of EPSs were performed using the phenol sulfuric acid method, as previously reported [81]. 

### 3.10. Characterization of the Monosaccharides of EPSs

The determinations of the monosaccharide of crude polysaccharides were analyzed by the HPLC method as previously described [82]. Analytical standards (HPLC purity ≥ 9%) of 6 monosaccharides, including arabinose (Ara), fucose (Fuc), galactose (Gal), glucose (Glc), amino-glucose (GlcN), and mannose (Man) were purchased from Merck (Shanghai, China). Qualitative analysis was performed by comparing the retention times and the peak areas with those of the standards. 

### 3.11. Evaluation of Bioflocculanting and MGP Bioactivities

Bioflocculating activity evaluations of the EPSs were performed according to the procedures previously reported [27,28,29,30]. The prepared EPSs were dissolved in the distilled water for furthering the bioactivity assay. The measurements using a kaolin clay suspension flocculation assay calculated as the bioflocculation rate (%) were used and performed using 96-well microplates with at least 6 replicates [27,28,29,30]. Microalgae growth-promoting (MGP) activity of bacterial strain LZ-28 toward algal strain LZT09 in a co-culture system was evaluated, as previously reported [83]. The preparation of the algal culture extract [29,30] and the effects on bacterial growth were analyzed by measuring the optical changes of bacterial density recorded at OD_600nm_ and performed in a SpectraMax M2 model 96-well microplate reader (Molecular Devices, LLC, San Jose, CA, USA). All the results are expressed as the means ± SD. The correlation coefficients of the monosaccharide portions with the bioflocculanting activities and the statistics significance were both performed using the Spearman’s rank order correlation analysis and plotted with the OriginPro (version 8.0) (OriginLab Corp., Northampton, MA, USA). A *p*-value of less than 0.05 was considered to be statistically significant for all analyses.

## Figures and Tables

**Table 1:** Phenotypic and genotypic characteristics of the bacterial strain LZ-28 and the other five *M. alba* strains.

Characteristic	LZ-28	JLT354-W^T^	KD53	L6M1-5	JL351	F15
Isolation source	Marine dinoflagellate	Seawater	Marine diatom	Deep-sea sediment	Surface seawater	Deep-sea sediment
Colony color	Light-Yellow	White	Yellow	Yellow–white	Yellow	Cream–yellow
Cell size (µm)	(0.7–1.0) × (2.0–2.9)	(0.7–0.9) × (2.0–3.2)	(0.5–1.0) × (2.2–2.9)	(0.7–0.8) × (1.0–2.0)	(0.5–0.8) × (0.7–1.5)	(0.7–1.2) × (1.6–3.4)
NaCl range (optimum, *w/v*, %)	1.0–10.0 (2.5)	1.0–10.0 (1.0–3.0)	1.0–6.0 (3.0)	0.5–15.0 (3.0–5.0)	0.5–9.0 (1.0–4.0)	0–10.0 (3.5)
pH range (optimum)	5.0–10.0 (7.0)	6.0–9.0 (8.0)	6.0–9.5 (7.5–8.0)	5.0–10.5 (7.0)	7.0–10.0 (8.0)	6.0–11.0 (7.0–8.0)
Temperature range (optimum, °C)	15–40 (25–28)	10–30 (25)	16–37 (28)	10–41 (28–30)	15–40 (30)	4–50 (35–37)
Oxidase activity	+	+	+	−	+	+
Catalase activity	+	+	−	+	+	+
Poly-β-hydroxybutyrate	+	+	−	−	+	−
API 20E test						
Citrate utilization	−	−	−	w	w	+
Gelatinase	+	+	−	+	+	+
API 20NE test						
Reduction of nitrite	+	+	+	+	−	+
Gelatin hydrolysis	+	+	−	+	+	+
Phenylacetic acid utilization	−	+	+	+	+	−
Polar lipid profile *^a^*	PC, DPG, PE	AL	PC, PE, AL	PE, AL	PC, DPG, PE, AL, GL	PC, PE, GL
Fatty acid profile *^b^*						
C_16:0_	3.2	5.7	5.3	4.8	5.3	4.5
C_17:0_	1.1	*tr*	1.0	*tr*	*tr*	ND
C_18:0_	7	9.2	11.1	10	8.6	8.5
C_12:1_ 3OH	4.7	3.3	2.8	3.1	3.2	3.1
C_17:1_ω8*c*	−	*tr*	*tr*	*tr*	*tr*	1
C_18:1_ω7c 11-methyl	10.8	6.6	7.9	7.5	6.1	5.4
C_19:0_ cyclo ω8c	3.2	5.7	5.3	4.8	5.3	4.5
Summed feature 8	65.5	70.8	69.5	70.7	72.5	74.3
16S rRNA gene similarity (%)	−	99.70	99.77	99.92	99.62	99.40
DNA G+C content (mol%) *^c^*	64.9	65.2	65.0	65.0	65.1	65.0
ANI value of LZ-28 (%)	−	98.0	98.2	98.0	98.0	97.9
AAI value of LZ-28 (%)	−	98.4	98.1	98.1	98.5	98.2
dDDH value of LZ-28 (%)	−	84.3	83.5	83.9	84.3	83.1

All data were obtained from this study unless otherwise indicated. +, positive; W, weakly positive; −, negative. *^a^* PC, phosphatidylcholine; DPG, diphosphatidylglycerol; PE, phosphatidylethanolamine; AL, aminolipids; GL, glycolipid. *^b^* Values represent percentage (%) of total fatty acid contents. *tr*, trace amount (less than 1.0%). ND, not detected. Summed feature 8 comprises C_18:1_ω7c and/or C_18:1_ω6c. *^c^* Data were calculated from the respective genome sequences.

**Table 2:** The isolation sources and general genomic features of the ten selected *Mameliella alba* strains in this study.

Strain	Isolation Source and Year	Genome Size (Mb)	G+C Content (mol%) *^a^*	Protein	CDS ^*b*^	GenBank Accession No.
LZ-28	Toxic marine dinoflagellate *Alexandrium catenella* LZT09, East China Sea, 2018	5.66	64.94	5502	5497	JAANYX000000000
KD53	Marine diatom *Phaeodactylum tricornutum,* Xiamen, China, 2013	5.42	65.10	5278	5223	NIWC00000000
Ep20	Marine dinoflagellate, *Symbiodinium* sp., 2015	5.51	65.04	5345	5343	QAEF00000000
PBVC088	Toxic marine dinoflagellate, *Pyrodinium bahamense* var. *Compressum*, 2012	5.51	64.96	5689	5632	LZNT00000000
UMTAT08	Marine dinoflagellate *Alexandrium tamiyavanichii* AcMS01, 1997	5.84	65.01	5761	5719	JSUQ00000000
JLT354-W^T^	Seawater, South China Sea, 2006	5.26	65.21	5126	5132	FMZI00000000
JL351	Surface seawater (111°00′ E, 20°59′ N), South China Sea, 2006	5.42	65.15	5290	5275	NIWA00000000
KU6B *^c^*	Surface seawater, Boso Peninsula, Japan (34.9° N, 134.89° E), 2011	5.83	64.95	6009	4886	AP022337-022340
L6M1-5	Deep-sea sediment (2835 m), South Atlantic Ocean (15.18° S, 13.88° W), 2011	5.90	64.96	5821	5783	NIVZ00000000
F15	Deep-sea sediment (7118 m, 141°59.7′ E, 10°59.7′ N), Western Pacific Ocean, 2015	5.79	64.95	5694	5589	NIWB00000000

*^a^* Data were calculated based on the respective genome sequences. *^b^* CDS, coding DNA sequence. *^c^* The complete genome contained a circular chromosome (AP022337) and three circular plasmids (AP022338/AP022339/AP022340) [34].

## Data Availability

All relevant data are within the manuscript.

## Supplementary Materials

The following are available online at https://www.mdpi.com/article/10.3390/md20050321/s1, Figure S1: Comparison of the average nucleotide identity (ANI), average amino acid identity (AAI), and digital DNA–DNA hybridization (dDDH) values based on the genomes of the ten M. alba strains. Figure S2: Comparison of the COG and KEGG categories among the ten selected M. alba strains. The functional genes of the core and unique groups distributed by COG database are shown in panes A and B, respectively. The panes C and D represent the distribution of the core and unique genes annotated by the KEGG database, respectively. The sizes of the circles in each pane are proportional to the numbers of the different COG or KEGG categories. The strains isolated from marine algae are marked with green.
