# Supplementary material for: Taxonomic and Bioactivity Characterizations of Mameliella alba Strain LZ-28 Isolated from Highly Toxic Marine Dinoflagellate Alexandrium catenella LZT09
Source: Mar Drugs. 2022 May 12;20(5):321. doi: 10.3390/md20050321 (PMC9147911; doi:10.3390/md20050321)
Supplement: Supplementary file 1 [file marinedrugs-20-00321-s001.zip › marinedrugs-1692039-supplementary.pdf]

Supplementary Materials

Taxonomic and Bioactivity Characterizations of Mameliella alba Strain LZ-28 Isolated from Highly Toxic Marine Dinoflagellate *Alexandrium catenella* LZT09

Cheng-Zhe Ren [1], Hui-Min Gao [1], Jun Dai [2], Wen-Zhuo Zhu [1],Fei-Fei Xu [3], Yun Ye [4], Xiao-Ling Zhang [1,5,*] and Qiao Yang [1,5,*]

[1] Department of Marine Chemistry, College of Marine Science and Technology, Zhejiang Ocean University, Zhoushan 316022, China; renchengzhe@zjou.edu.cn (C.-Z.R.); gaohuimin@zjou.edu.cn (H.-M.G.);

zhuwenzhuo@zjou.edu.cn (W.-Z.Z.)

[2] Natural "111" Center for Cellular Regulation and Molecular Pharmaceutics, Key Laboratory of Fermentation Engineering, Ministry of Education, College of Bioengineering, Hubei University of Technology, Wuhan 430068, China; jundai@hbut.edu.cn

[3] Zhejiang Yimeiyuan Testing Technology Co., Ltd., Zhoushan 316022, China; smexu713@163.com

[4] Zhoushan Natural Resources Surveying and Mapping Design Centre, Zhoushan 316021, China; smeye918@gmail.com

[5] ABI Group, Zhejiang Ocean University, Zhoushan 316022, China

* **Correspondences**: zhangxiaoling@zjou.edu.cn (X.-L.Z.); qiaoyang1979@whu.edu.cn (Q.Y.)

Figure S1. Comparison of the average nucleotide identity (ANI), average amino acid identity (AAI) and digital DNA-DNA hybridization (dDDH) values based on the genomes of the ten *M. alba* strains.

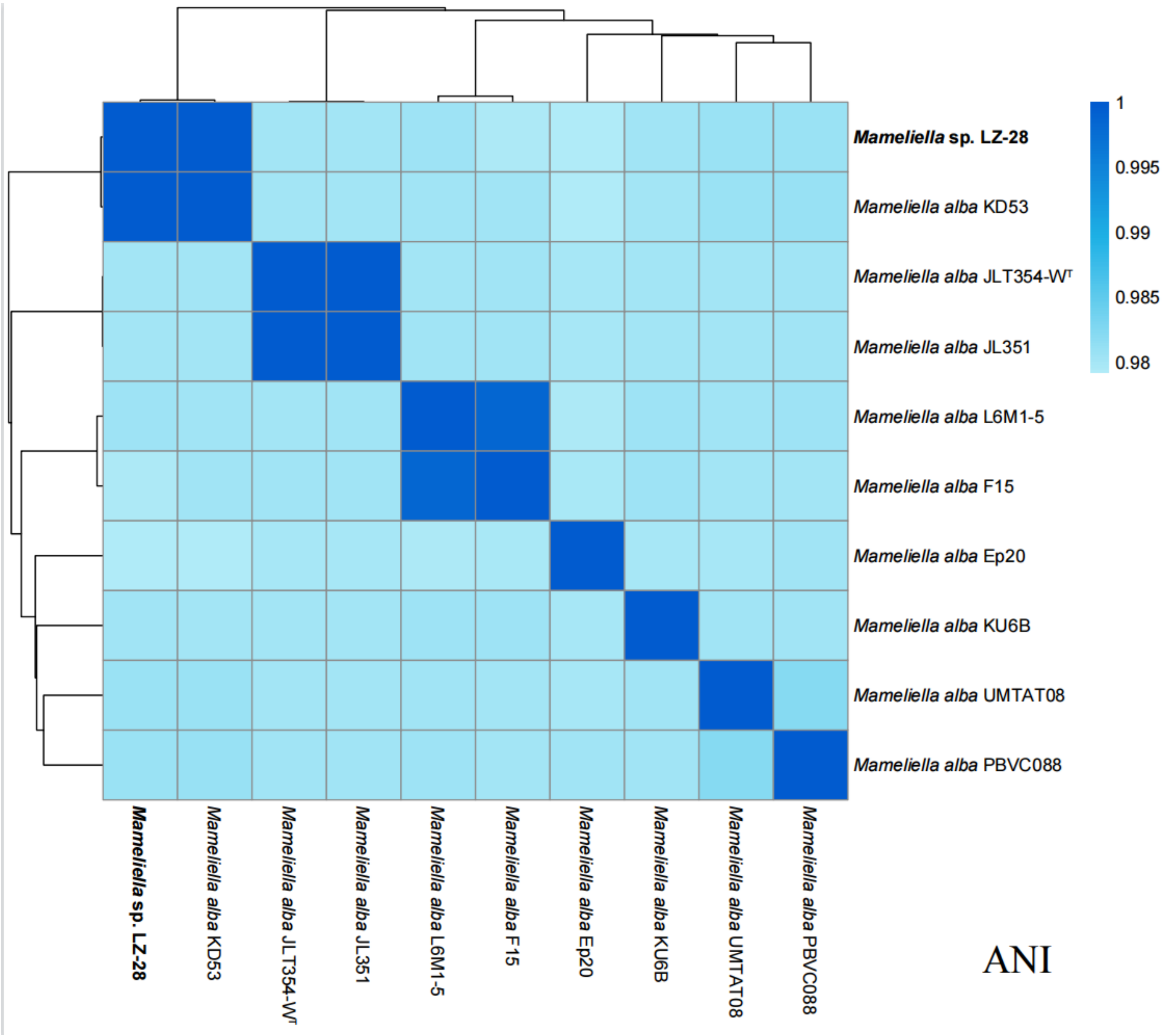

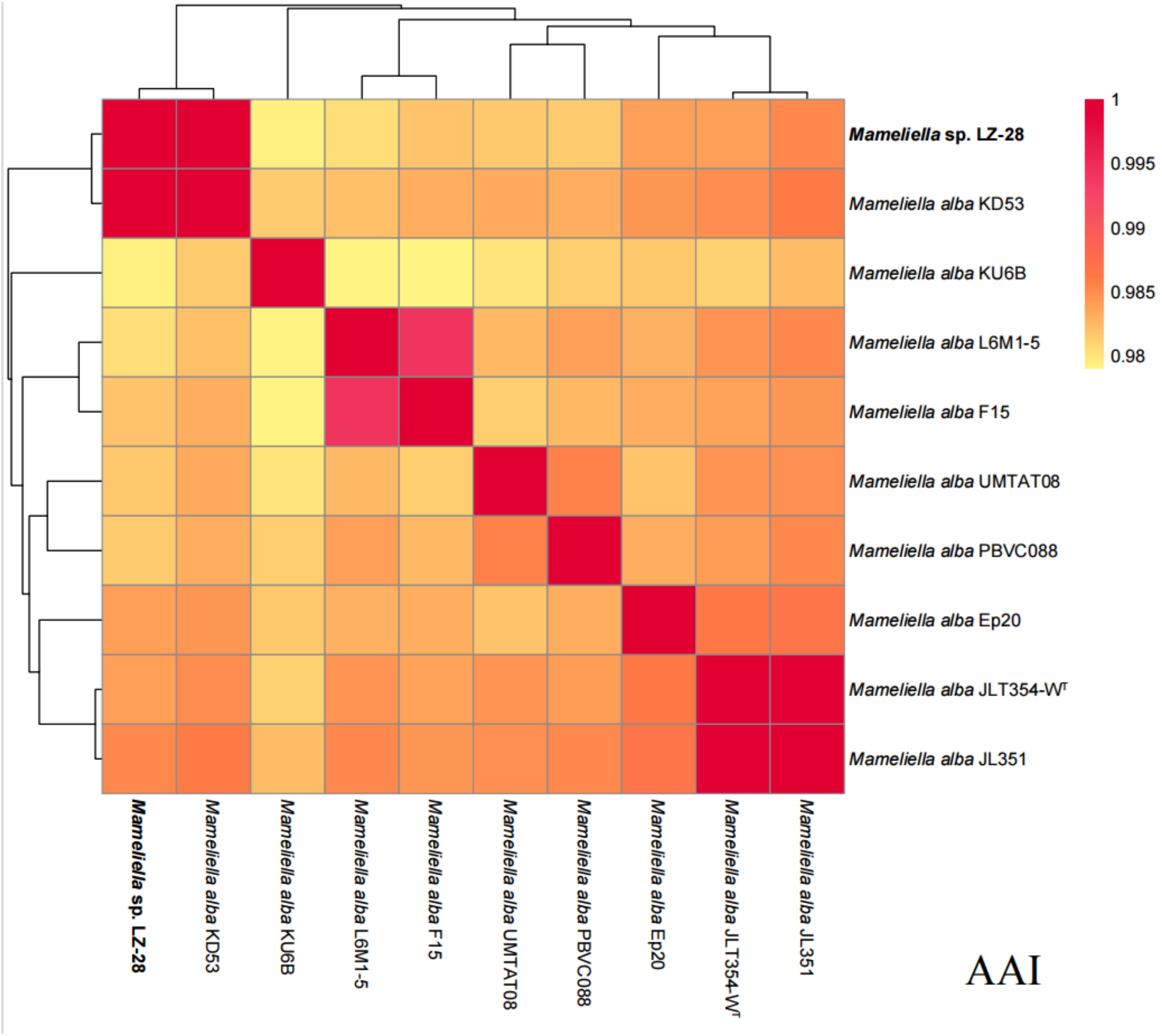

Mameliella sp. LZ-28
Mameliella alba KD53
Mameliella alba KU6B
Mameliella alba L6M1-5
Mameliella alba F15
Mameliella alba UMTAT08
Mameliella alba PBVC088
Mameliella alba Ep20
Mameliella alba JLT354-W^T
Mameliella alba JL351
1
0.995
0.99
0.985
0.98
AAI

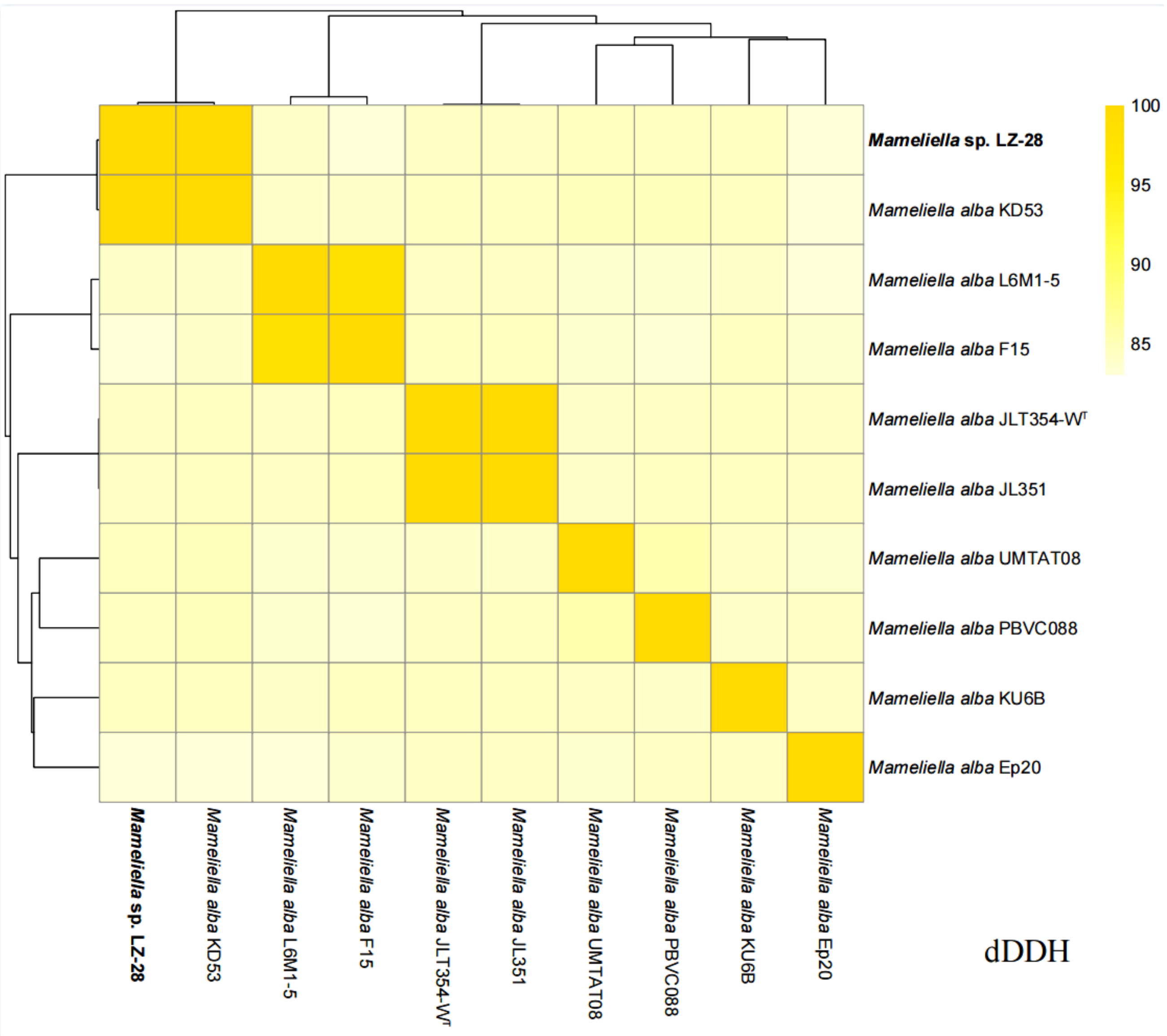

100
95
90
85
Mameliella sp. LZ-28
Mameliella alba KD53
Mameliella alba L6M1-5
Mameliella alba F15
Mameliella alba JLT354-W^T
Mameliella alba JL351
Mameliella alba UMTAT08
Mameliella alba PBVC088
Mameliella alba KU6B
Mameliella alba Ep20
dDDH

Figure S2. Comparison of the COG and KEGG categories among the ten selected *M. alba* strains. The functional genes of the core and unique groups distributed by COG database were shown in pane A and B, respectively. The pane C and D represented the distribution of the core genes and unique genes annotated by KEGG database, respectively. The sizes of the circles in each pane were proportional to the numbers of the different COG or KEGG categories. The strains isolated from marine algae were marked with green.

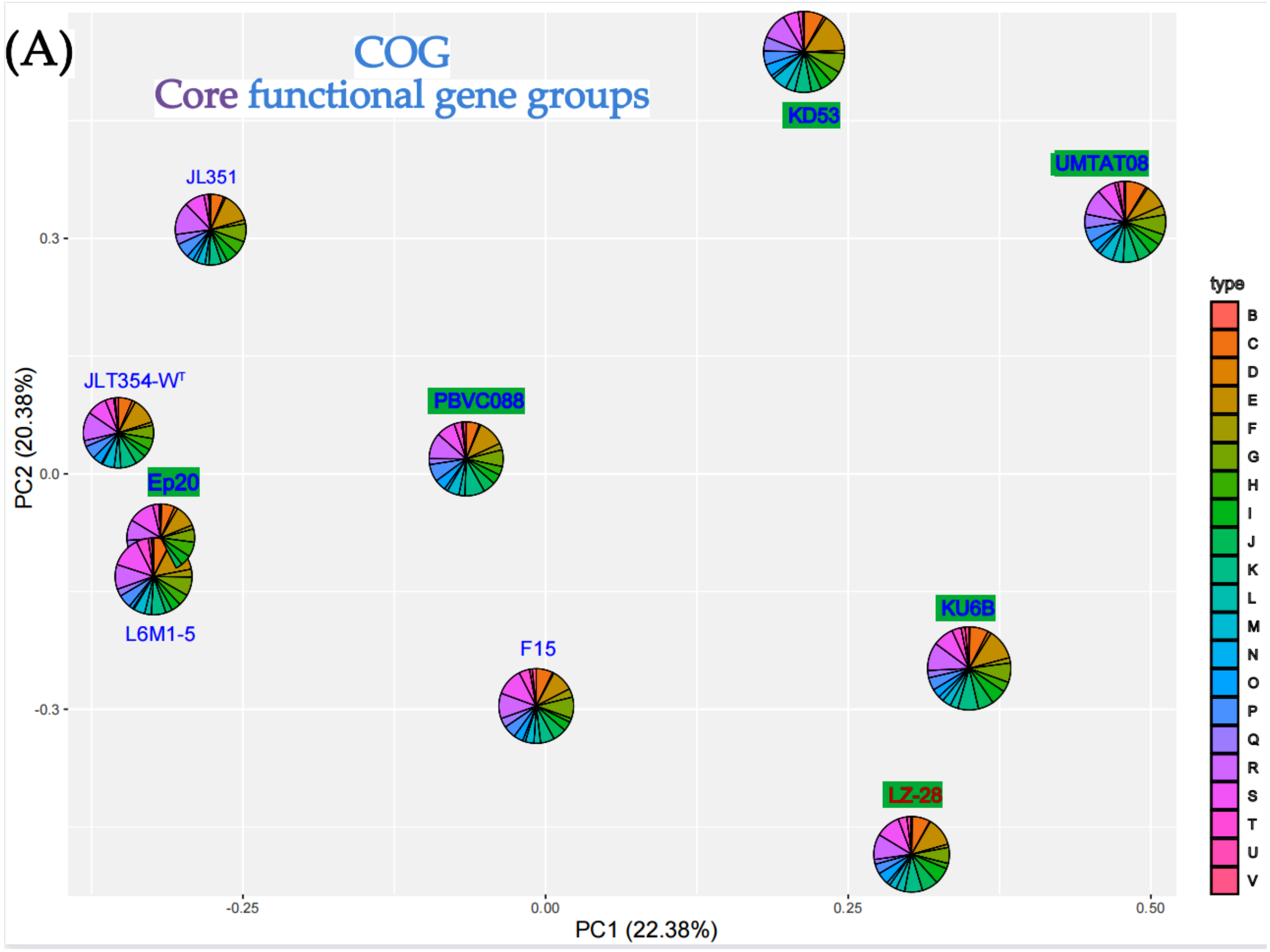

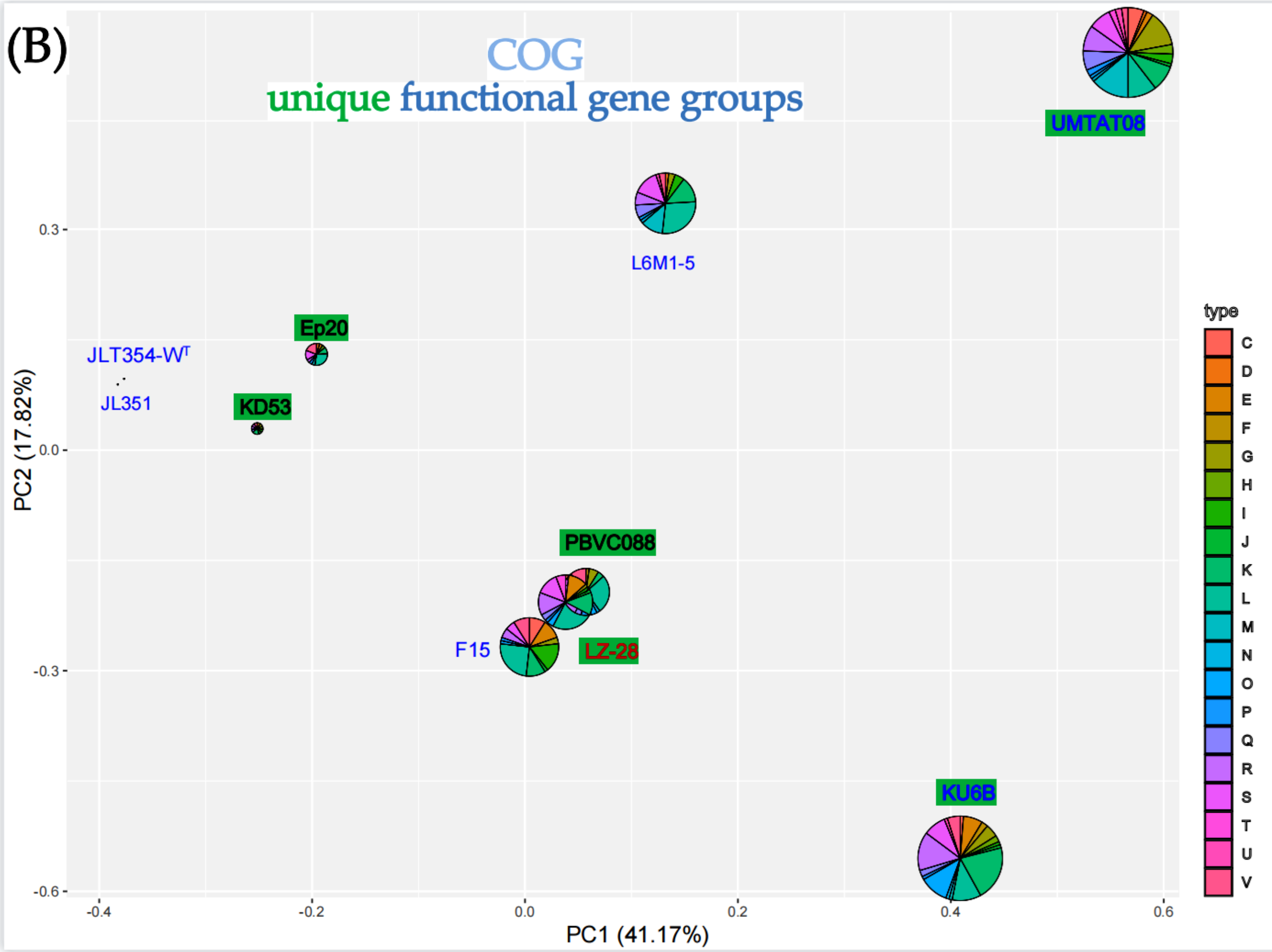
(B)
COG
unique functional gene groups
UMTAT08
L6M1-5
Ep20
JLT354-W^T
JL351
KD53
PBVC088
F15
LZ-28
KU6B
PC2 (17.82%)
PC1 (41.17%)
0.3
0.0
-0.3
-0.6
-0.4
-0.2
0.0
0.2
0.4
0.6
type
C
D
E
F
G
H
I
J
K
L
M
N
O
P
Q
R
S
T
U
V

(C)

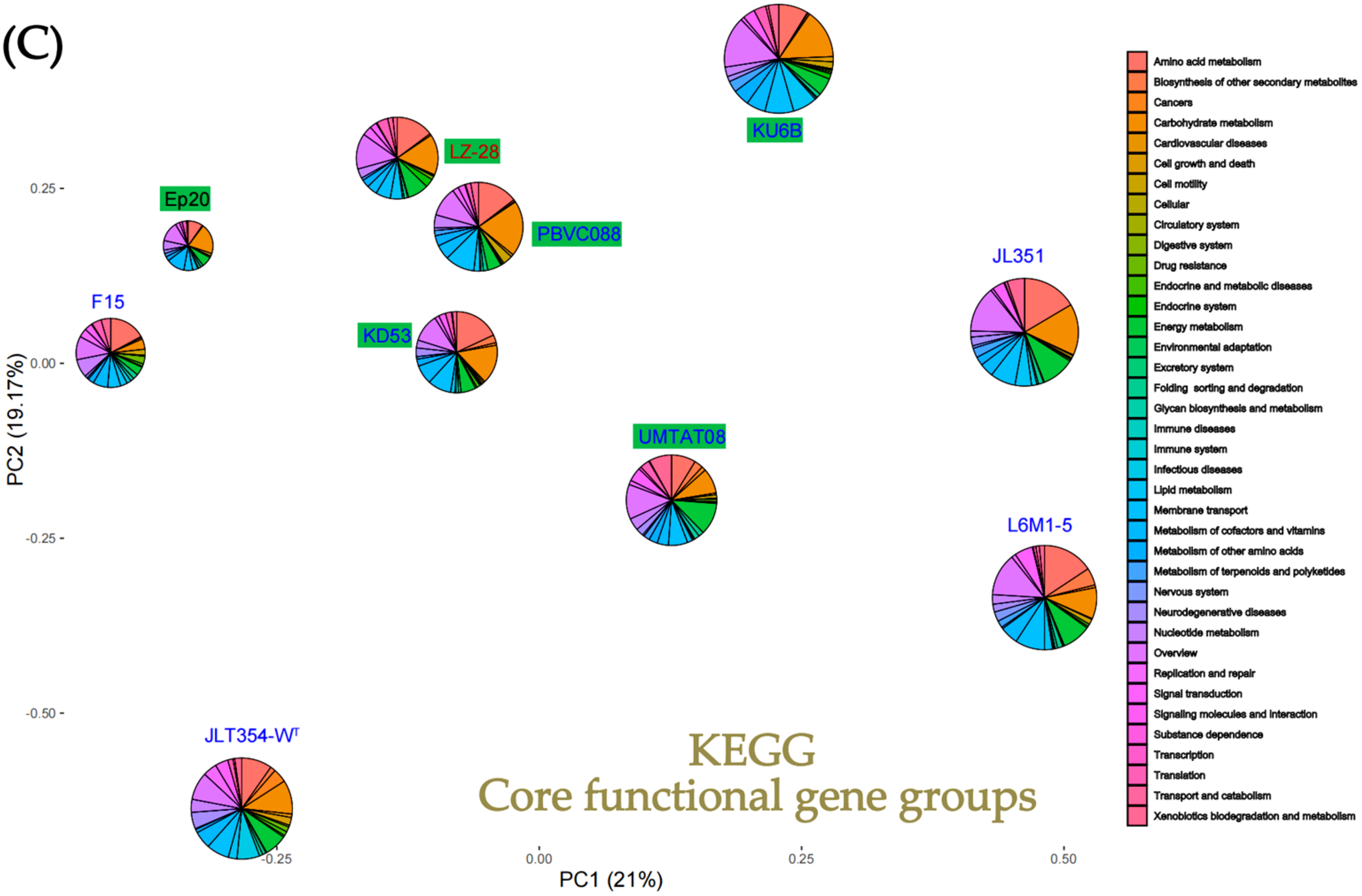

KU6B
LZ-28
Ep20
PBVC088
JL351
F15
KD53
UMTAT08
L6M1-5
JLT354-W[T]
0.25
0.00
-0.25
-0.50
PC2 (19.17%)
-0.25
0.00
0.25
0.50
PC1 (21%)
KEGG
Core functional gene groups
Amino acid metabolism
Biosynthesis of other secondary metabolites
Cancers
Carbohydrate metabolism
Cardiovascular diseases
Cell growth and death
Cell motility
Cellular
Circulatory system
Digestive system
Drug resistance
Endocrine and metabolic diseases
Endocrine system
Energy metabolism
Environmental adaptation
Excretory system
Folding sorting and degradation
Glycan biosynthesis and metabolism
Immune diseases
Immune system
Infectious diseases
Lipid metabolism
Membrane transport
Metabolism of cofactors and vitamins
Metabolism of other amino acids
Metabolism of terpenoids and polyketides
Nervous system
Neurodegenerative diseases
Nucleotide metabolism
Overview
Replication and repair
Signal transduction
Signaling molecules and interaction
Substance dependence
Transcription
Translation
Transport and catabolism
Xenobiotics biodegradation and metabolism

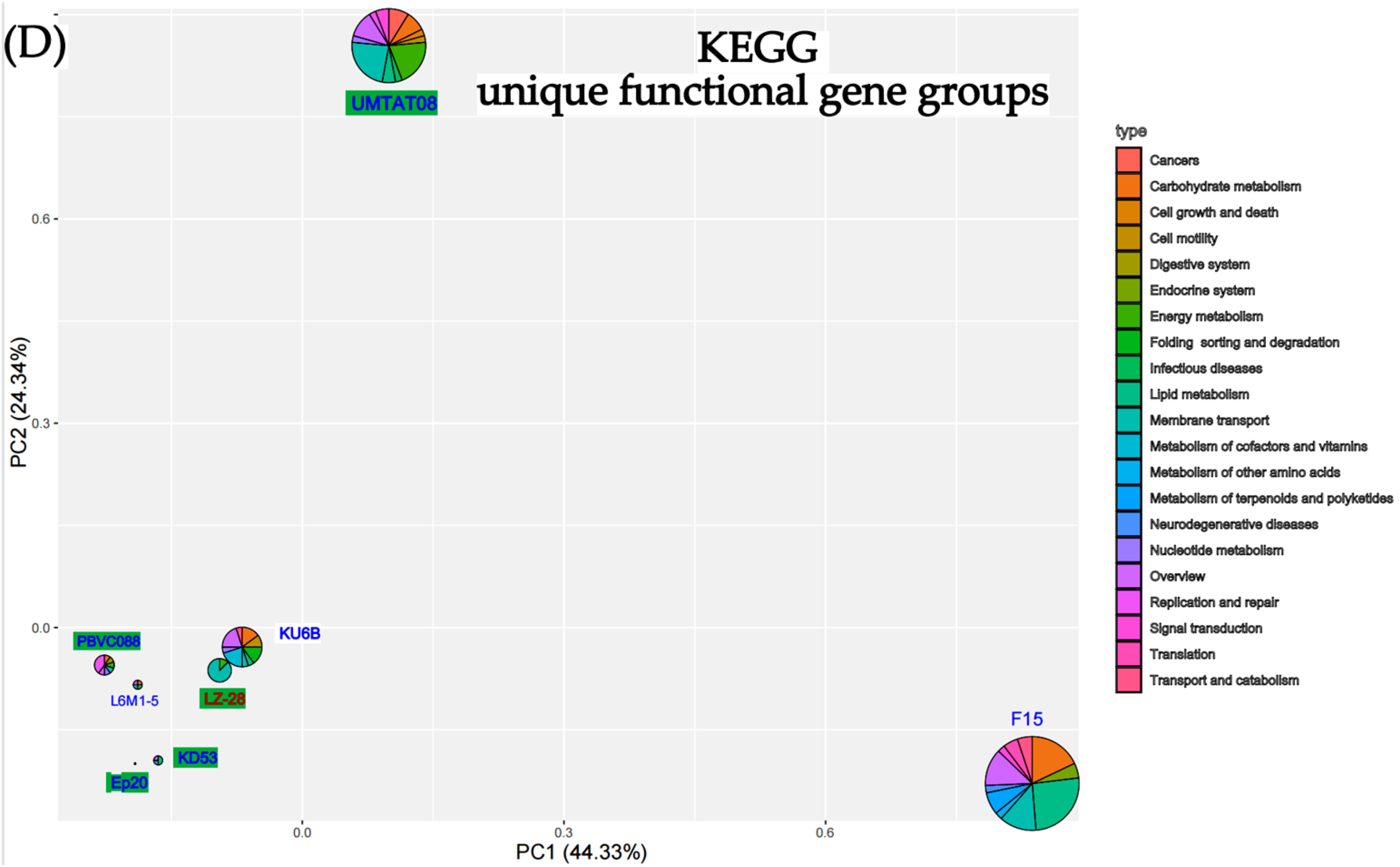

(D)
KEGG
unique functional gene groups
UMTAT08
KU6B
PBVC088
L6M1-5
LZ-28
KD53
Ep20
F15
0.6
0.3
0.0
PC2 (24.34%)
0.0
0.3
0.6
PC1 (44.33%)
type
Cancers
Carbohydrate metabolism
Cell growth and death
Cell motility
Digestive system
Endocrine system
Energy metabolism
Folding sorting and degradation
Infectious diseases
Lipid metabolism
Membrane transport
Metabolism of cofactors and vitamins
Metabolism of other amino acids
Metabolism of terpenoids and polyketides
Neurodegenerative diseases
Nucleotide metabolism
Overview
Replication and repair
Signal transduction
Translation
Transport and catabolism
